# Kidney involvement in leptospirosis: a systematic review and meta-analysis

**DOI:** 10.1007/s15010-025-02492-1

**Published:** 2025-03-20

**Authors:** Astha Sethi, Tirlangi Praveen Kumar, Kutty Sharada Vinod, Carl Boodman, Rachana Bhat, Prithvishree Ravindra, Souvik Chaudhuri, Seema Shetty, V. Shashidhar, Attur Ravindra Prabhu, Nitin Gupta

**Affiliations:** 1 Department of Infectious Diseases, Kasturba Medical College, Manipal, Manipal Academy of Higher Education, Manipal, 576104 India; 2 Department of Medicine, All India Institute of Medical Sciences, Manglagiri, India; 3 Department of Clinical Sciences, Institute of Tropical Medicine, Antwerp, Belgium; 4 University of Antwerp, Antwerp, Belgium; 5 Department of Emergency Medicine, Kasturba Medical College, Manipal, Manipal Academy of Higher Education, Manipal, 576104 India; 6 Department of Critical Care Medicine, Kasturba Medical College, Manipal, Manipal Academy of Higher Education, Manipal, 576104 India; 7 Department of Microbiology, Kasturba Medical College, Manipal, Manipal Academy of Higher Education, Manipal, 576104 India; 8 Department of Nephrology, Kasturba Medical College, Manipal, Manipal Academy of Higher Education, Manipal, 576104 India

**Keywords:** Leptospirosis, Acute kidney injury, Chronic kidney disease, Dialysis, Oliguria

## Abstract

**Introduction:**

From a public health perspective, it is essential to understand the burden of kidney involvement in leptospirosis. We aimed to assess the frequency of acute kidney injury (AKI) and chronic kidney disease (CKD) in patients with leptospirosis.

**Methodology:**

This systematic review and meta-analysis included all articles up to 14.08.2024 from three databases (PubMed, Scopus, Web of Science) using search terms related to leptospirosis and kidney involvement. After de-duplication, two independent reviewers independently checked the articles in two phases (title-abstract and full-text), and a third reviewer adjudicated any conflicts. Patient demographics, diagnostic procedures, and details of kidney involvement were extracted from the included studies. Risk of bias analysis was done using the Joanna Briggs Institute critical appraisal tool. A random effects model estimated the pooled rates for AKI, oliguria, and the need for dialysis.

**Results:**

Of the 5913 retrieved articles, 48 met the eligibility criteria. The pooled incidence of AKI, reduced urine output, and dialysis requirement was 49.2% (95%CI: 38.2-60.2%, I^2^ of 99.4%), 31.5% (95%CI: 24.2-38.7%, I^2^-96.1%) and 14.4% (95%CI: 10.3-18.4%, I^2^-97%) respectively. The pooled mean serum creatinine and urea levels at admission were 3.6 mg/dl (95% CI: 2.9–4.2, I^2^-99.1%) and 131.8 mg/dl (95% CI: 98.7-164.9, I^2^-98.6%), respectively. In four studies, the incidence of new-onset CKD after leptospirosis infection varied from 13 to 62%.

**Conclusion:**

AKI reduced urine output and the requirement for dialysis are frequent complications in patients with leptospirosis. Increased resources for their management in endemic areas are essential to mitigate the burden.

**Supplementary Information:**

The online version contains supplementary material available at 10.1007/s15010-025-02492-1.

## Introduction

Leptospirosis is a zoonotic bacterial infection caused by a spirochaete (*Leptospira* spp) [1]. It is one of the most common febrile illnesses in the tropics [2]. The bacterium commonly affects the renal tubules of several mammals (rodents, dogs, cattle, etc.) and is excreted in the urine, contaminating water or soil [1]. Humans acquire leptospirosis when exposed to contaminated water or soil. The exposure can be occupational, recreational, or during severe weather events. Leptospirosis is estimated to cause close to a million cases and 60,000 deaths annually [3]. The disease is distributed worldwide, with most reports from South America, Oceania and South Asia [3, 4]. It presents as a febrile illness, with a percentage of affected individuals developing renal, hepatic, pulmonary or neurological complications [1, 4].

Kidney injury in leptospirosis results from a combination of infection-related immune dysregulation, metabolic disturbances, and clinical factors [5]. Understanding the complex kidney-related complications of leptospirosis is vital for effective prevention, management, and resource allocation in endemic regions [5]. Acute Kidney Injury (AKI), with or without decreased urine output, is among the most common complications in patients with leptospirosis [2, 5, 6]. Leptospires colonise the proximal tubule and trigger an immune response, leading to tubulointerstitial nephritis [5]. Several inflammatory cytokines/biomarkers, like Neutrophil gelatinase-associated lipocalin (NGAL) and Kidney injury molecule (KIM-1), that are associated with renal damage are expressed at high levels in the kidney in leptospirosis [7, 8]. Some admitted patients with leptospirosis also require dialysis due to severe kidney injury. Persistent infection and kidney inflammation can lead to fibrosis and chronic kidney disease (CKD) [9]. Although the kidney function of leptospirosis cases, except urinary concentration, recovered by six months in the study by Daher et al., subsequent studies show that AKI in leptospirosis can progress to chronic kidney disease (CKD) as well [10–12]. A previous systematic review of leptospirosis studies published before 2017 focussed on mortality outcomes in AKI and included only 23 studies [13]. They did not study CKD as an outcome or perform meta-analyses to pool the outcomes [13]. The main objective is, therefore, to assess the frequency of AKI and CKD in patients with a confirmed microbiological diagnosis of leptospirosis.

## Methodology

### Registration

The systematic review and meta-analysis (SRMA) was registered with PROSPERO (Registration number- CRD42024583461) and is reported according to the PRISMA guidelines.

### Research question

What is the frequency of AKI and CKD in adult leptospirosis patients? (Table 1).

**Table 1:** Research question, search string and eligibility criteria for inclusion of studies

PICOS	Description	Search String	Inclusion criteria	Exclusion Criteria
Population	Leptospirosis patients	(leptospira OR leptospirosis OR weil*)	Microbiologically diagnosed	Clinically diagnosed
Intervention/ Control	Not applicable			
Outcome	AKI, CKD	(“Acute kidney injury” OR “chronic kidney disease” OR “kidney failure” OR “renal insufficiency”)	AKI or CKD reported	Incomplete or missing data
Studies			RCT, observational studies (*n* > 50)	Case reports, conference abstracts, reviews

*Abbreviations*: AKI: Acute Kidney Injury, CKD- Chronic Kidney Disease, RCT- Randomised controlled trial

### Search strategy

A systematic bibliographic search was conducted on PubMed/MEDLINE, Scopus, and Web of Science. A search string was created by combining the keywords related to ‘population’ and ‘outcome’. Additionally, MeSH index terms were added in the PubMed Search String (Table 1). The citations of the included articles were searched for any additional papers that could be included.

### Article screening and selection

After deleting duplicates, the titles and abstracts of retrieved articles were independently screened by two reviewers (AS and TPK). The full text of the included articles was retrieved and evaluated independently for eligibility by two reviewers. A third reviewer (NG) resolved any discrepancy between the two reviewers. A validated web tool (rayyan.ai) facilitated the screening process. All studies published up to the 14th of August 2024 were included. The eligibility criteria have been summarised in Table 1. Articles published in all languages were included.

### Data extraction (selection and coding)

Data from the adult population were extracted wherever possible. Kidney involvement was reported for the entire population for studies that did not report adult data separately. Two reviewers independently extracted the publication details, study setting, average age, number of patients with leptospirosis, and the method for diagnosing leptospirosis. The method of diagnosis was recorded as molecular (polymerase chain reaction assay in blood or urine), serological (microscopic agglutination test, enzyme-linked immunosorbent assay, or immunochromatography) or both. The definition of kidney involvement (AKI, CKD, decreased urine output, dialysis), number of patients with kidney involvement, and mean serum urea and creatinine at admission were also recorded. If mean and standard deviation for creatinine and urea were unavailable, they were estimated from the median and inter-quartile range using procedures delineated by Wan et al. [14]. The data were recorded in a Microsoft Excel spreadsheet (Microsoft Corporation, version 2501).

### Risk of bias (quality) assessment

The risk of bias was assessed using the JBI critical appraisal tool for observational study [15].

### Data synthesis

The pooled frequency of AKI, decreased urine output, and dialysis requirement were calculated. A subgroup analysis was performed for the pooled frequency of AKI according to the regions. Sensitivity analyses were performed separately for studies that included patients of all severity levels and those limited to inpatients. The pooled mean serum urea and creatinine at admission were also calculated. All analyses were conducted with a random effects model (Der Simonian and Laird) using the R statistical software (R 3.5.3 GUI 1.70 El Capitan build 7632) and an open-source meta-analysis software developed by Wallace et al. [16]. The heterogeneity among the eligible articles was assessed using the I² index.

## Results

### Inclusion of studies

**Fig. 1:**
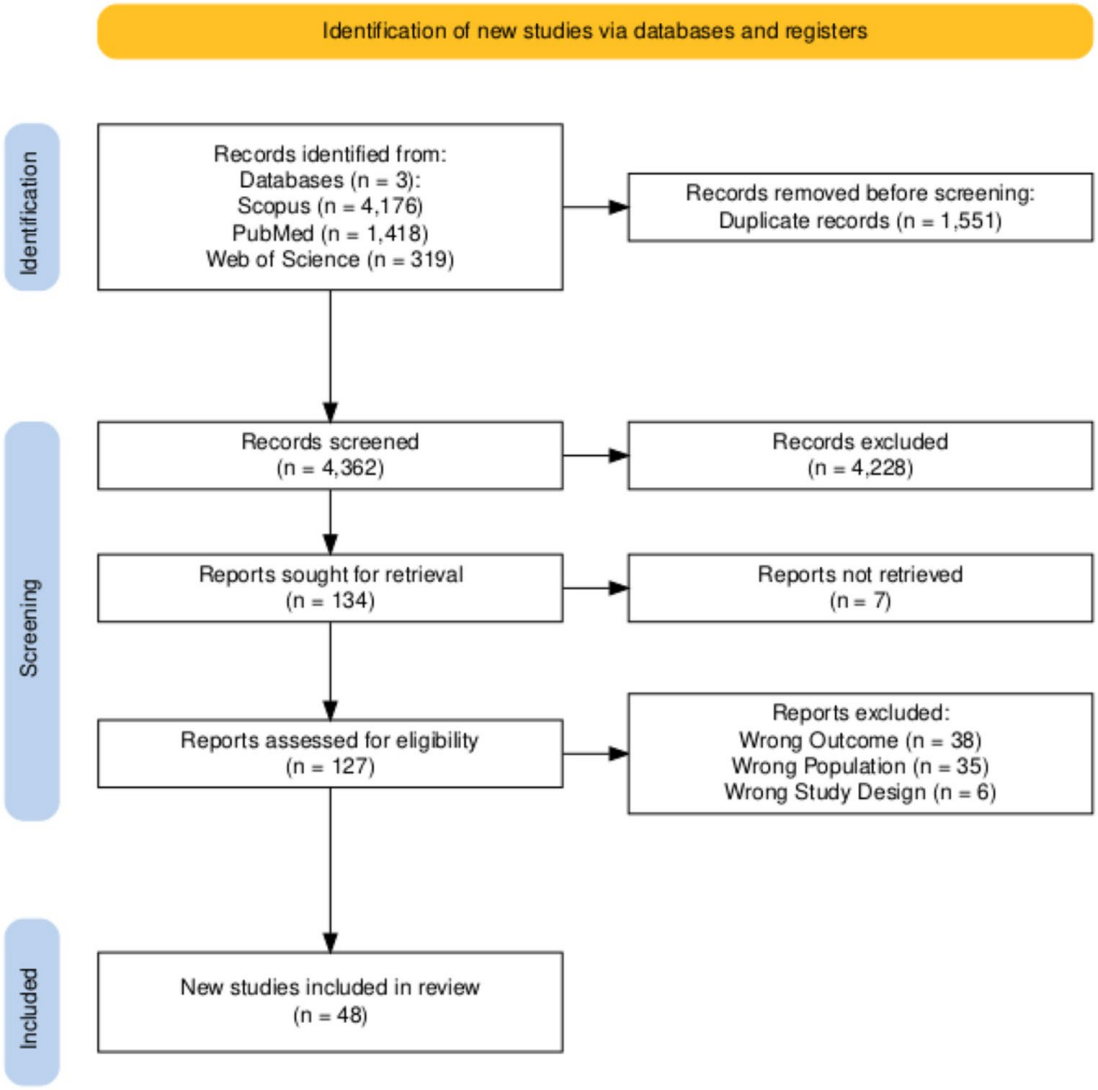
PRISMA flow chart showing screening and inclusion of studies with leptospirosis and kidney involvement. *Note*: Records not retrieved imply that the full text of this article could not be retrieved despite an extensive search. Reports excluded imply that the articles were excluded after the title-abstract screening or full-text review because they did not meet the eligibility criteria

A total of 5913 articles were retrieved from three databases. After the title-abstract and full-text screening, 48 studies were included in the final analysis. Of these, 44 studies reported data on only AKI, two on only CKD, and two reported both AKI and CKD (Fig. 1).

### AKI

Seven of the 44 studies reporting AKI originated from the same centre in Brazil and included overlapping patient data from 1985 to 2017 [17–23]. To avoid overlapping data in the SRMA, the latest study that included the whole period of 1985–2017 was included for further analysis [21]. The 40 studies on AKI were conducted across various countries, with most studies from South Asia [India (*n* = 11), Sri Lanka (*n* = 5)], Southeast Asia [Thailand (*n* = 5), Malaysia (*n* = 2), Indonesia (*n* = 1)], and South America [Brazil (*n* = 3), Cuba (*n* = 1)]. The average age of the patients ranged commonly between 30 and 50 years (Table 2). Across the included studies, most patients were male, with proportions ranging commonly from 60 to 90% (Table 2). The definitions of AKI and oliguria varied across the studies (Supplementary Table 1). Only 12 studies applied standardised criteria given by AKIN (Acute Kidney Injury Network), RIFLE (Risk, Injury, Failure, Loss and End-stage Kidney Disease) or KDIGO (Kidney Disease: Improving Global Outcomes) for diagnosis of AKI, which may impact comparability and reliability of reported AKI frequencies (Supplementary Table 1). Of the 15 studies that reported oliguria, 10 used the definition of < 0.5 ml/kg/hour of urine for more than 6 h (Supplementary Table 1). The remaining studies used an oliguria cut-off of less than 400–500 ml /day.

**Table 2:** Summary of included studies on adult patients with acute kidney injury

Sn	Author name	Country	Year of publication	Type of Study	Sample size	Age group	Method of diagnosis	Mean (SD) Age	Number Male (%)
1	Arumugam et al. [6]	India	2016	Prospective	67	All Ages	Serology	29 (16)	48 (71.6)
2	Bharadwaj et al. [24]	India	2002	Prospective	74	All Ages	Serology	ND	ND
3	Borse et al. [25]	India	2023	Prospective	52	Adult	Serology	ND	40 (76.9)
4	Bourrier et al. [26]	France	1988	Retrospective	99	All Ages	Serology	46 (19)	83 (83.8)
5	Carrasco et al. [27]	Cuba	1991	Prospective	215	Unclear (Probably adults)	Serology or molecular	ND	ND
6	Chang et al. [11]	Taiwan	2022	Retrospective	2145	Adult	Serology or molecular	48.99 (15)	1497 (69.7)
7	Christova et al. [28]	Bulgaria	2003	Retrospective	455	Unclear (Probably adults)	Serology	45.55 (11)	414 (90.9)
8	Daher et al. [21]	Brazil	2019	Retrospective	507	All Ages	Serology	ND	419 (82.6)
9	Dassanayake et al. [29]	Sri Lanka	2012	Prospective	62	Unclear (Probably adults)	Serology	39 (19)	47 (75.8)
10	Fonseka et al. [30]	Sri Lanka	2023	Prospective	88	All Ages	Serology or molecular	47(16)	78 (88.6)
11	Goswami et al. [31]	India	2014	Retrospective	101	All Ages	Serology or culture	41 (16)	ND
12	Hariri et al. [32]	Malaysia	2022	Retrospective	525	Adult	Serology	38(17)	344 (65.5)
13	Herath et al. [33]	Sri Lanka	2019	Prospective	128	Unclear (Probably adults)	Serology	46 (14)	107 (83.5)
14	Holla et al. [34]	India	2018	Retrospective	202	All Ages	Serology	40 (15)	142 (70.2)
15	Ittyachen et al. [35]	India	2007	Retrospective	53	Adult	Serology	ND	40 (75.4)
16	Junior et al. [36]	Brazil	2011	Retrospective	287	All Ages	Serology	37 (16)	232 (80.8)
17	Majumdar et al. [37]	India	2013	Retrospective	77	All Ages	Serology	ND	49 (63.6)
18	Markum et al. [38]	Indonesia	2004	Retrospective	68	All Ages	Serology	38	51 (75)
19	Muthusethupathi et al. [39]	India	1995	Prospective	57	Adult	Serology	40	50 (87.7)
20	Nair et al. [40]	India	2016	Prospective	151	Adult	Serology	ND	ND
21	Nisansala et al. [41]	Sri Lanka	2021	Prospective	79	Unclear (Probably adults)	Serology or molecular	45 (16)	69 (87.3)
22	Nisansala et al. [42]	Sri Lanka	2019	Retrospective	108	Unclear (Probably adults)	Serology	44 (16)	ND
23	Niwattayakul et al. [43]	Thailand	2009	Retrospective	148	All Ages	Serology	ND	107 (72.2)
24	Olszyna et al. [44]	Netherlands	1998	Retrospective	159	Unclear (Probably adults)	Serology	ND	ND
25	Panaphut et al. [45]	Thailand	2002	Prospective	121	All Ages	Serology	38 (13)	114 (94.21)
26	Parmar et al. [46]	India	2016	Prospective	84	All Ages	Serology or molecular	38.78 (15)	54 (64.2)
27	Perić et al. [47]	Croatia	2005	Retrospective	270	All Ages	Serology	37.4 (10)	230 (85.1)
28	Pertuiset et al. [48]	Réunion	1988	Retrospective	249	Adult	Serology		
29	Phannajit et al. [49]	Thailand	2023	Prospective	217	Unclear (Probably adults)	Serology or molecular	47 (17)	180 (82.9)
30	Philip et al. [50]	Malaysia	2021	Prospective	83	All Ages	Serology or molecular	42 (18)	54 (65)
31	Raoult et al. [51]	France	1983	Retrospective	60	Unclear (Probably adults)	Serology or molecular	ND	ND
32	Rista et al. [52]	Albania	2022	Prospective	119	All Ages	Serology	49 (15)	109 (91.5)
33	Sethi et al. [53]	India	2010	Retrospective	86	All Ages	Serology	32.6 (1)	49 (56.9)
34	Smith et al. [54]	Australia	2019	Retrospective	55	Unclear (Probably adults)	Serology or molecular	47 (32–62)	49 (89)
35	Srisawat et al. [55]	Thailand	2015	Prospective	113	Unclear (Probably adults)	Serology or molecular	43 (13)	35 (30.9)
36	Sukmark et al. [56]	Thailand	2018	Prospective	105	Unclear (Probably adults)	Serology or molecular	43 (15)	86 (81.9)
37	Teles et al. [57]	Brazil	2016	Retrospective	205	Adult	Serology	35.4 (13)	171 (83.4)
38	Vidigal et al. [58]	Spain	2014	Retrospective	86	All Ages	Serology	43.1 (14)	73 (84.8)
39	Wang et al. [59]	Taiwan	2018	Retrospective	57	Unclear (Probably adults)	Serology	59 (16)	42 (73.6)
40	Yersin et al. [60]	Seychelles	1998	Prospective	75	All Ages	Serology or molecular	35.5 (16)	63 (84)

*Abbreviation*: Sn- Serial number, SD- standard deviation, ND- No data in the reported study

**Fig. 2:**
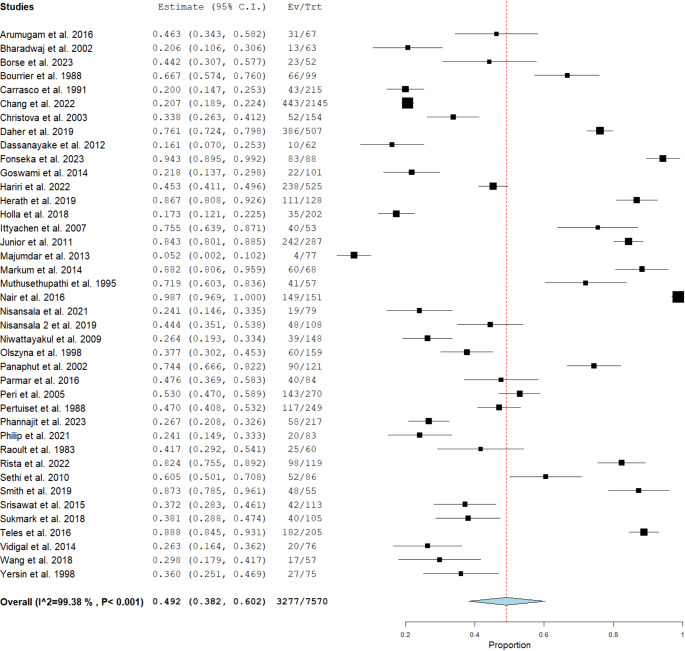
Pooled frequency of acute kidney injury in patients with leptospirosis

The pooled frequency of AKI in patients with leptospirosis was 49.2% (95%CI: 38.2-60.2%, I^2^ of 99.4%) (Table 3) (Fig. 2). The pooled frequency varied across regions, with South America (Brazil, Cuba) showing the highest pooled proportion (**67.4%**), followed by Europe (**48.8%**), South Asia (India, Sri Lanka) (**48.3%**), and Southeast Asia (Thailand, Malaysia, Indonesia) (**45.1%**). All regions exhibited high heterogeneity (Supplementary Figs. 1–4). There was no significant improvement in heterogeneity after removing the three studies that only included patients with severe disease [Frequency- 46.3% (95%CI: 34.8-57.7%), I^2^-99.4%] (Supplementary Fig. 5) or after removing four studies that included both inpatient and outpatients [Frequency- 51% (95%CI: 39.4-62.5%), I^2^-99.4%] (Supplementary Fig. 6). A subgroup analysis was done to include only those patients where a standardised definition (AKIN, RIFLE, KDIGO) for AKI was used; the heterogeneity remained high [62.4% (95%CI: 47.1-77.7%), I^2^-99.2%] (Supplementary Fig. 7).

The pooled frequency of decreased urine output was 31.5% (95%CI: 24.2-38.7%, I^2^-96.1%) (Supplementary Fig. 8). The pooled frequency of dialysis requirement was 14.4% (95%CI: 10.3-18.4%, I^2^-97% (Supplementary Fig. 9). The pooled frequency of mortality in the included studies was 8.8% (95%CI: 7.3-10.3%, I^2^-77.5%) (Supplementary Fig. 10). Mortality in patients with AKI was 12.5% (95%CI: 8.4-16.7%, I^2^-90.1%) (Supplementary Fig. 11). The pooled mean serum creatinine and urea levels were 3.6 mg/dl (95% CI: 2.9–4.2, I^2^-99.1%) and 131.8 mg/dl (95% CI: 98.7-164.9, I^2^-98.6%), respectively (Supplementary Figs. 12 and 13).

**Table 3:** Kidney involvement and outcomes of the patients with leptospirosis in the included studies

Sn	Author name	Sample size	Admission	Severity	Standardised definition	AKI (%)	Decreased UO (%)	Dialysis (%)	Overall mortality (%)	Mortality AKI (%)
1	Arumugam et al. [6]	67	IP	All Severity	No	31 (46.2)	22 (32.8)	17 (25.4)	3 (4.4)	2 (6.4)
2	Bharadwaj et al. [24]	74	IP	All Severity	No	13 (17.6)	21 (28.4)	ND	11 (14.8)	ND
3	Borse et al. [25]	52	IP	All Severity	No	23 (44.2)	14 (26.9)	ND	ND	ND
4	Bourrier et al. [26]	99	IP	All severity	No	66 (66.7)	ND	16 (16.2)	8 (8)	ND
5	Carrasco et al. [27]	215	IP	All Severity	No	43 (20.0)	43 (20)	24 (11.2)	18 (8.3)	15 (34.8)
6	Chang et al. [11]	2145	IP	All Severity	No	443 (20.7)	ND	77 (3.6)	182 (8.4)	46 (10.3)
7	Christova et al. [28]	154	IP	All Severity	No	52 (33.8%)	ND	ND	30 (6.5)	ND
8	Daher et al. [21]	507	IP	All Severity	Yes	386 (76.1)	127 (25)	199 (39.3)	75 (14.7)	ND
9	Dassanayake et al. [29]	62	IP	All Severity	Yes	10 (16.1%)	20 (32.3)	ND	ND	ND
10	Fonseka et al. [30]	88	IP	Severe	No	83 (94.3)	65 (73.9)	ND	15 (17)	ND
11	Goswami et al. [31]	101	IP	All Severity	No	22 (21.8)	35 (34.7)	22 (21.8)	17 (16.8)	6 (27.2)
12	Hariri et al. [32]	525	IP	All severity	Yes	238 (45.3)	ND	42 (8.0)	34 (6.4)	27 (11.3%)
13	Herath et al. [33]	128	IP	All Severity	Yes	111 (86.7)	100 (78.1)	ND	ND	36 (32.4)
14	Holla et al. [34]	202	IP	All Severity	No	35 (17.3)	52 (25.7)	ND	7 (3.4)	ND
15	Ittyachen et al. [35]	53	IP	Severe	No	40 (75.5)	ND	5 (9.4)	2 (3.7)	ND
16	Junior et al. [36]	287	IP	All Severity	Yes	242 (84.3)	55 (19.2)	105 (36.6)	ND	37 (15.2)
17	Majumdar et al. [37]	77	IP/OP	All Severity	No	4 (5.2)	ND	ND	ND	ND
18	Markum et al. [38]	68	IP	All Severity	No	60 (88.2)	ND	ND	2 (2.9)	ND
19	Muthusethupathi et al. [39]	57	IP	All Severity	No	41 (71.9)	31 (54.4)	23 (40.4)	2 (3.5)	ND
20	Nair et al. [40]	151	IP	All Severity	Yes	149 (98.7)	ND	ND	16 (10.5)	ND
21	Nisansala et al. [41]	79	IP	All Severity	Yes	19 (24.1)	13 (16.5)	ND	ND	2 (10.5)
22	Nisansala et al. [42]	108	IP	All Severity	No	48 (44.4%)	28 (25.9%)	3 (2.8%)	5 (4.6%)	ND
23	Niwattayakul et al. [43]	148	IP	All Severity	No	39 (26.3%)	ND	ND	5 (14.7)	2 (2.35)
24	Olszyna et al. [44]	159	IP	All Severity	No	60 (37.7)	ND	ND	8 (5.03)	ND
25	Panaphut et al. [45]	121	IP	All Severity	No	90 (74.4)	26 (21.5)	3 (2.5)	17 (14)	1 (1.1)
26	Parmar et al. [46]	84	IP	All Severity	No	40 (47.6)	40(47.6)	ND	ND	ND
27	Perić et al. [47]	270	IP	All Severity	No	143 (53.0)	ND	19 (7.0)	ND	ND
28	Pertuiset et al. [48]	249	IP	All Severity	No	117 (47.0)	54 (21.7)	ND	33 (13.2)	2 (1.7)
29	Phannajit et al. [49]	217	IP	All severity	Yes	58 (26.7)	ND	2 (0.9)	30 (13.8)	12 (20.6)
30	Philip et al. [50]	83	IP	All severity	No	20 (24.1)	ND	ND	11 (12.5)	ND
31	Raoult et al. [51]	60	IP/OP	All Severity	No	25 (41.7)	ND	ND	4 (6.6)	ND
32	Rista et al. [52]*	119	IP	All Severity	Yes	98 (82.4)	18 (15.1)	ND	10 (8.4)	10 (10.2)
33	Sethi et al. [53]	86	IP/OP	All severity	No	52 (60.5)	25 (29.1)	ND	5 (5.8)	ND
34	Smith et al. [54]	55	IP	Severe	Yes	48 (87.2)	35 (63.6)	ND	2 (3.6)	2 (4.1)
35	Srisawat et al. [55]	113	IP	All Severity	No	42 (37.2)	ND	10 (8.8)	ND	ND
36	Sukmark et al. [56]	105	IP	All Severity	No	40 (38.1)	ND	ND	ND	ND
37	Teles et al. [57]	205	IP	All Severity	Yes	182 (88.8)	10 (4.9)	47 (22.9)	19 (9.2)	18 (9.8)
38	Vidigal et al. [58]	76	IP/OP	All severity	No	20 (26.3)	ND	ND	6 (6.9)	ND
39	Wang et al. [59]	57	IP	All severity	Yes	17 (29.8)	4 (7)	4 (7.0)	11 (19.2)	5 (29.4)
40	Yersin et al. [60]	75	IP	All Severity	No	27 (36%)	ND	8 (10.7)	6 (8)	ND

Abbreviation: Sn- Serial number, AKI: Acute kidney injury, UO- urine output, IP- inpatient, OP- outpatient, ND- No data

### CKD

Four observational studies from Asia investigated new-onset CKD in leptospirosis patients [11, 12, 49, 61] (Table 4). Except for one study that did not define CKD, the other three used standardised definitions given by KDIGO [61]. Since renal tubules are the primary site of involvement of leptospirosis, Phannajit et al. included tubulopathy (phosphate and magnesium wasting) in the absence of reduced glomerular filtration as CKD as well [49]. The proportion of new-onset CKD ranged from 13 to 62%, likely reflecting differences in CKD definitions, study design, follow-up duration, and geographic factors. Due to the limited number of studies and inconsistencies in CKD definitions, methodology, and follow-up duration, the incidence of CKD was not pooled.

**Table 4:** Summary of included studies that reported chronic kidney disease

Sn	Author name	Country	Year of publication	Type of Study	Sample size (*n*)	Method of diagnosis	CKD definition	Number with CKD [*n* (%)]	Average follow-up (years)
1	Chang et al. [11]	Taiwan	2022	Retrospective	2145	Serology or Molecular	KDIGO	282 (13.1)	4.3
2	Fatema et al. [61]	India	2023	Retrospective	386	Serology	Not Defined	240 (62.1)	NA
3	Phannajit et al. [49]	Thailand	2023	Prospective	83	Serology or Molecular	KDIGO*	33 (39.7)	4.3
4	Yang et al. [12]	Taiwan	2015	Cross-sectional study	1034	Serology	KDIGO	231 (22)	No follow-up

Abbreviation: Sn- Serial number, CKD- chronic kidney disease

### Critical appraisal of literature

Most studies (84%) clearly defined their subject populations and settings, ensuring contextual relevance (Supplementary Table 2). An objective standard criterion used to diagnose leptospirosis was present in 89% of the patients. However, only 75% of studies explicitly stated their inclusion criteria, and 70% employed robust statistical analyses, indicating potential gaps in methodological rigour. A minority of studies had unclear reporting of inclusion criteria and statistical methods, potentially introducing bias or limiting reliability. Two criteria—identification of confounding factors and strategies to address them—were excluded, as they were irrelevant to this SRMA. Outcome measurement criteria for AKI and CKD are discussed in their respective sections and were omitted from Supplementary Tables 2 to avoid redundancy.

## Discussion

Leptospirosis, a zoonotic disease prevalent in low- and middle-income countries, poses significant challenges to healthcare systems due to its frequent association with AKI and its potential to progress to CKD. Kidney involvement was a common manifestation of leptospirosis in this SR, with a pooled AKI frequency of nearly 50%. With a pooled frequency of 31.5%, decreased urine output was a common manifestation, potentially reflecting the severity of renal involvement in leptospirosis-related AKI. The high proportion of complications (dialysis requirement: 14%) and poor outcomes (mortality: 8%) highlight the need for resource allocation in endemic areas. Notably, mortality in patients with AKI (12.5%) was significantly higher than the overall pooled mortality (8.8%), suggesting that AKI worsens prognosis in leptospirosis patients. The elevated pooled mean serum creatinine (3.6 mg/dL) and urea (131.8 mg/dL) suggest substantial renal dysfunction, further emphasising the severity of AKI in leptospirosis patients. Beyond the acute phase, leptospirosis significantly contributes to CKD, with incidences varying from 13 to 62% in our review. This challenges the traditional notion of fully reversible kidney injury in leptospirosis.

Our SR included studies that focussed on adult patients with leptospirosis. Only one study had data on paediatric patients as well. They noted that the incidence of AKI was higher in adult patients when compared to paediatric patients [17]. Renal involvement in leptospirosis can range from mild biochemical abnormalities to severe disease, resulting in oliguria, the requirement for dialysis and death [62, 63]. The incidence of AKI varied between the studies from less than 10% to more than 90%, resulting in significant statistical heterogeneity. The wide variation could be the difference in the virulence of pathogenic serovars, definitions for AKI used in the study, and the severity of the studied population. Only 12 of 40 studies defined AKI using RIFLE, AKIN, and KDIGO classifications (Supplementary Table 1). Some studies used serum creatinine cut-offs alone to define AKI (Supplementary Table 1). The frequency of AKI increased to 62% when a subgroup analysis was done to include only those studies that reported AKI using standardised definitions, but the heterogeneity remained high. In those studies that used standardised definitions, it is unlikely that the choice of criteria (RIFLE, AKIN or KDIGO) would have led to significant differences in the diagnosis of AKI. In the study by Junior et al., the frequency of AKI with RIFLE and AKIN criteria were very similar (82% and 84%, respectively).

In our meta-analysis, 9/40 studies reported AKI in over 80% of cases. Of these, three explicitly mentioned the inclusion of severe cases only, explaining the high frequency of AKI [30, 35, 54]. In the subgroup analysis, there was no significant reduction in heterogeneity after removing the studies that included only severe disease. The remaining six studies also had some degree of bias towards the selection of more severe patients, potentially leading to an overestimation of AKI prevalence [33, 36, 38, 40, 52, 57]. Ideally, the pooled frequency of AKI should have been calculated separately for mild and severe cases; however, most studies did not provide this stratification. Herath et al. had a very high percentage of pulmonary haemorrhage (62.5%), indicating that the cohort consisted primarily of patients with severe disease [33]. Markum et al. used a creatinine cut-off of more than 1.5 mg/dl to define AKI [38]. Since they relied on a single cut-off, they would not have been able to exclude patients with pre-existing kidney disease before leptospirosis infection. This could have led to an overestimation of the incidence. In the study by Nair et al. that reported AKI in different tropical fevers, the reported frequency of AKI in other diseases was also very high [E.g. Dengue: 59/85 (69%)], suggesting some referral bias [40]. In the study by Teles et al., of the diagnosed cases of leptospirosis, 40% of cases were excluded from analysis due to lack of clinical data [57]. It is possible that blood investigations were not carried out in the mild cases, leading to their exclusion and false elevation of AKI frequency. In the study by Rista et al., patients admitted to the nephrology department were included, creating a possibility of selection bias [52].

Although non-oliguric AKI is common, approximately 30% of AKI cases in our review presented with reduced urine output. Few studies defined reduced urine output; even in these studies, the cutoffs varied widely (Supplementary Table 1). In our review, the dialysis requirement was high (14%), indicating the need for improving access to dialysis machines in resource-limited endemic settings. A South American study demonstrated that early, daily dialysis significantly reduces mortality in leptospirosis patients [64]. The mortality rate for leptospirosis patients with AKI (12.5%) was higher than the overall mortality rate (8.8%) in our SR, highlighting the severe impact of kidney involvement on clinical outcomes. In several of the included studies, AKI, oliguria, and dialysis requirements were independent predictors of mortality [13, 23, 36, 50, 65]. In the study by Daher et al. (2016), the worst AKI stages had higher mortality [23]. The mean levels of urea and creatinine were also found to be higher in non-survivors [23].

**Fig. 3:**
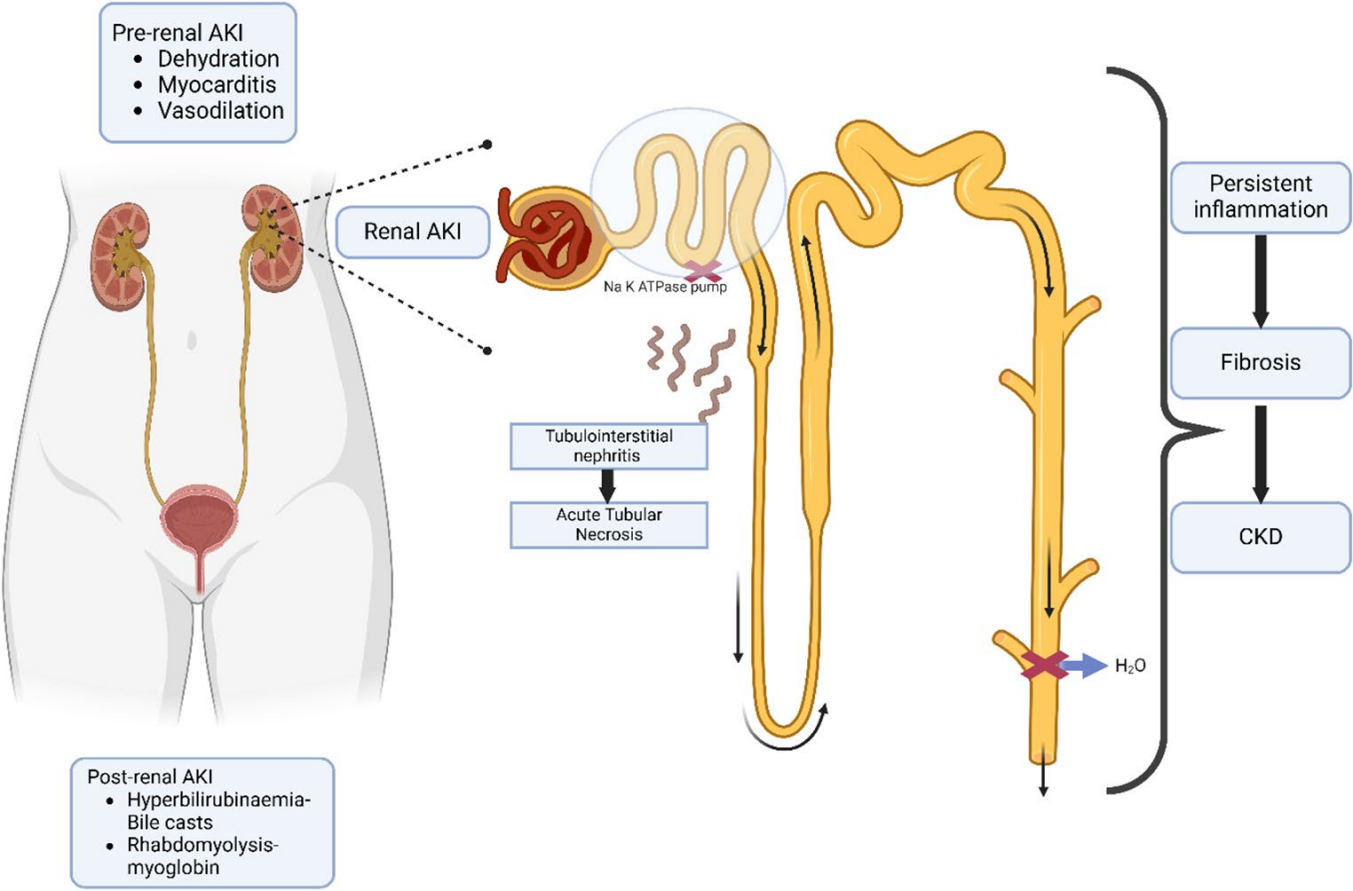
Pathophysiology and mechanisms of acute kidney injury and chronic kidney disease in leptospirosis. *Abbreviation*: AKI- acute kidney injury, CKD- chronic kidney disease. Renal involvement in leptospirosis can be due to pre-renal, post-renal and renal causes. The bacteria reaches the proximal convoluted tubules and blocks the Na-K ATPase. There is simultaneous resistance of medullary collecting tubules to vasopressin. This leads to non-oliguric tubulo-interstitial nephritis. This may progress to oliguric acute tubular necrosis. Persistent inflammation can lead to fibrosis and chronic kidney disease

Several mechanisms contribute to AKI in leptospirosis, which is often reversible but may sometimes progress to irreversible damage [20]. We provide a simplified overview of AKI pathophysiology in leptospirosis, acknowledging its complexity and incomplete understanding (Fig. 3). Following inoculation, the bacteria rapidly colonise the kidneys, a unique feature of leptospiral infection [66]. In the kidney interstitium, leptospiral lipopolysaccharides and outer membrane proteins trigger inflammation, resulting in tubulointerstitial nephritis [5, 62, 66]. Leptospires preferentially colonise the proximal tubules, as evidenced by tubular dysfunction (glycosuria, bicarbonaturia), even without AKI [62]. Rista et al. noted that tubular dysfunction preceded and predicted AKI in patients with leptospirosis [52]. Some studies have reported simultaneous resistance of medullary collecting tubules to vasopressin at this stage, resulting in the absence of oliguria [67]. At the initial stage of leptospirosis, paradoxical hypokalaemia is common. Brazilian researchers extensively studied leptospiral GLP-1, which inhibits Na-K ATPase in renal tubular cells, leading to hypokalemia [66]. In those where inflammation is unregulated, this can progress to acute tubular necrosis, leading to oliguria. AKI can also result from thrombotic microangiopathy or glomerulonephritis due to direct endothelial damage caused by leptospires [62]. Prerenal AKI may result from dehydration, myocarditis, or vasodilation [62]. Bile casts (due to hyperbilirubinemia) or myoglobin casts (due to rhabdomyolysis) can obstruct renal tubules, causing cast nephropathy and oliguria [62]. The AKI can also be aggravated by hypotension, myocarditis, and nephrotoxic antibiotics [5]. High positive end-expiratory pressure (PEEP) can impair renal perfusion, while hypoxia and ischemic injury exacerbate AKI.

Although AKI in leptospirosis is often reversible, persistent spirochete-induced inflammation or immune dysregulation can lead to fibrosis and tubular atrophy (5,10). Loss of functional glomeruli leads to compensatory hyperfiltration in remaining nephrons, which may accelerate CKD progression. Persistent renal injury, driven by immune dysregulation, tubulointerstitial inflammation, and compensatory hyperfiltration, accelerates CKD progression [5]. Differences in study design, definitions, sample size, follow-up duration, and diagnostic methods may explain variations in CKD prevalence. Yang et al. conducted a cross-sectional survey in a highly endemic area of Taiwan, testing patients for leptospira serology and serum creatinine to estimate GFR. They found a higher CKD prevalence in seropositive patients than in seronegative ones (22% vs. 17%) [12]. They also included a prospective cohort of 88 patients exposed to floods in a highly endemic region and followed them for two years. They reported that the decrease in GFR was more pronounced in those who had persistently higher serological titres on follow-up [12]. The study by Fatema et al. from India, which reported a high incidence of CKD (62%), was a retrospective cohort study where patients diagnosed with leptospirosis over 10 years in a high endemic area were contacted to note whether they developed CKD or not [61]. The authors did not mention the mean years of follow-up or the definition of CKD. Interestingly, the healthy controls matched for age, gender and area also had a high incidence of CKD (20%) [61].

Studies included in the SR showed that most cases of CKD in leptospirosis happen in patients who have AKI, especially the ones who require dialysis. In the retrospective cohort study by Chang et al., the incidence of CKD in patients without AKI, patients with AKI (not requiring dialysis), and those requiring dialysis was 7%, 34% and 47%, respectively [11]. In the study by Phannajit et al., severe leptospirosis was more commonly associated with new-onset CKD than non-severe leptospirosis [49]. While the studies included in the SR focussed on symptomatic leptospirosis, it needs to be further seen whether asymptomatic colonisation in kidneys can also lead to CKD. In a study from a hyper-endemic area in Peru, close to 5% of patients with asymptomatic serological positivity due to leptospirosis had persistent shedding of the bacteria in urine [63]. While the authors did not study the incidence of CKD in that study, ongoing leptospiral shedding in the urine may also perpetuate inflammation and renal damage [5]. In a study conducted in Brazil on dogs, asymptomatic urine shedding was associated with chronic kidney disease [68]. Although the four studies in the SR observed the association between leptospirosis and CKD, establishing a direct causal relationship is challenging. Patients at risk for leptospirosis often reside in hot and humid climates, where factors such as dehydration, exposure to pesticides, high salt intake, and analgesic abuse are prevalent. These environmental and lifestyle factors can contribute to the progression of chronic kidney disease, making it challenging to attribute kidney dysfunction solely to leptospirosis. The multifactorial nature of chronic kidney disease in these individuals further complicates the identification of Leptospira as the primary causative agent. Renal involvement in leptospirosis may be associated with a significant burden on healthcare systems in endemic areas. Limited access to dialysis and critical care services exacerbates the impact of AKI, while the long-term burden of CKD strains resources further. Long-term follow-up programs are essential to monitor renal function and implement interventions to slow CKD progression.

Our review has a few limitations. High heterogeneity (I²>90%) across studies reflects variability in the type of study (prospective vs. retrospective), geographical regions, study settings (inpatient vs. outpatients), method of diagnosis, severity at presentation, and the definition used for kidney involvement. Indirect estimation of means and standard deviations for some creatinine and urea values may have introduced bias. Most studies used serological tests to diagnose leptospirosis, which is limited by cross-reactivity and undermines any attempt to analyse renal complications based on different *Leptospira* species.

These findings highlight the need for early detection and comprehensive management of leptospirosis-related kidney disease. Improving access to diagnostic tools and standardised AKI and CKD criteria is critical to optimising outcomes. Increased resources for intensive care and dialysis in endemic areas are essential to mitigate the burden of renal complications and improve patient prognosis.

## Electronic supplementary material

Below is the link to the electronic supplementary material.

Supplementary Material 1

## Data Availability

The data is available at reasonable request from the corresponding author.
