# Supplementary material for: Kidney involvement in leptospirosis: a systematic review and meta-analysis
Source: Infection. 2025 Mar 20;53(3):785–96. doi: 10.1007/s15010-025-02492-1 (PMC12137426; doi:10.1007/s15010-025-02492-1)

**Supplementary Table 1: Definitions of kidney involvement in the included studies**

| **Sn** | **Author name** | **Sample size** | **RIFLE** | **AKIN** | **KDIGO** | **Creatinine cut-off** | **Urine output definition** |
| --- | --- | --- | --- | --- | --- | --- | --- |
| 1 | Daher et al.(21) | 507 |  |  | Yes |  | <0.5 mL/kg/h |
| 2 | Dassanayake et al.(29) | 62 | Modified |  |  |  | <0.5L/day |
| 3 | Fonseka et al.(30) | 88 |  |  |  |  | <0.5 ml/kg/h |
| 4 | Goswami et al.(31) | 101 |  |  |  |  | <0.5L/day |
| 5 | Hariri et al.(32) | 525 |  | Yes |  |  | <400 ml/day |
| 6 | Herath et al.(33) | 128 |  |  | Yes |  | <0.5 mL/kg/h |
| 7 | Ittyachen et al.(35) | 53 |  |  |  | 1.4 |  |
| 8 | Junior et al. (36) | 287 |  | Yes |  |  | <0.5 mL/kg/h |
| 9 | Markum et al.(38) | 68 |  |  |  | 1.5 |  |
| 10 | Nair et al.(40) | 151 |  |  | Yes |  | <0.5 mL/kg/h |
| 11 | Nisansala et al.(41) | 79 |  |  | Yes |  | <0.5 mL/kg/h |
| 12 | Panaphut et al.(45) | 121 |  |  |  | 2 | < 400 ml/day |
| 13 | Phannajit et al.(49) | 217 |  |  | Yes |  | <0.5 mL/kg/h |
| 14 | Raoult et al.(51) | 60 |  |  |  | 2 |  |
| 15 | Rista et al.(52) | 119 | Yes | Yes |  |  | <0.5 mL/kg/h |
| 16 | Sethi et al.(53) | 86 |  |  |  | 1.4 |  |
| 17 | Smith et al.(54) | 55 |  |  | Yes |  | ≤500ml/day |
| 18 | Teles et al.(57) | 205 |  |  | Yes |  | <0.5 mL/kg/h |
| 19 | Vidigal et al.(58) | 86 |  |  |  | 2 |  |
| 20 | Wang et al.(59) | 57 |  |  | Yes |  | <0.5 mL/kg/h |

**Supplementary Table 2: Critical appraisal of the included studies**

| **Sn** | **Study Author** | **Study year** | **Inclusion criteria** | **Subject and setting** | **Exposure measurement** | **Measurement of condition** | **Statistical analysis** |
| --- | --- | --- | --- | --- | --- | --- | --- |
| 1 | Arumugam et al. | 2016 | No | Yes | Yes | Yes | Yes |
| 2 | Bharadwaj et al. | 2002 | Unclear | Unclear | Yes | Unclear | No |
| 3 | Borse et al. | 2023 | Yes | Yes | Yes | Yes | Yes |
| 4 | Bourrier et al. | 1988 | Yes | Yes | Yes | Yes | Yes |
| 5 | Carrasco et al. | 1991 | No | No | Yes | Yes | Unclear |
| 6 | Chang et al. | 2022 | Yes | Yes | Yes | Yes | Yes |
| 7 | Christova et al. | 2003 | Unclear | Yes | Yes | Yes | Unclear |
| 8 | Daher et al. | 2019 | Yes | Yes | Yes | Yes | Yes |
| 9 | Dassanayake et al. | 2012 | Yes | Yes | Yes | Yes | Yes |
| 10 | Fonseka et al. | 2023 | Yes | Yes | Yes | Yes | Yes |
| 11 | Goswami et al. | 2014 | Yes | Yes | Yes | Unclear | Yes |
| 12 | Hariri et al. | 2022 | Yes | Yes | Yes | Yes | Yes |
| 13 | Herath et al. | 2019 | Yes | Yes | Yes | Yes | Yes |
| 14 | Holla et al. | 2018 | Yes | Yes | Yes | No | Yes |
| 15 | Ittyachen et al. | 2007 | Yes | Yes | Yes | Yes | Unclear |
| 16 | Junior et al. | 2011 | Yes | Yes | Yes | Yes | Yes |
| 17 | Majumdar et al. | 2013 | Yes | Yes | Yes | Yes | Unclear |
| 18 | Markum et al. | 2004 | Yes | Yes | Yes | Yes | Yes |
| 19 | Muthusethupathi et al. | 1995 | No | Yes | Yes | Yes | Yes |
| 20 | Nair et al. | 2016 | Yes | Yes | Yes | Yes | Unclear |
| 21 | Nisansal et al. | 2021 | Yes | Yes | Yes | Yes | Yes |
| 22 | Nisansala et al. | 2019 | Yes | Yes | Yes | Yes | Yes |
| 23 | Niwattayakul et al. | 2009 | Unclear | Yes | Yes | Yes | No |
| 24 | Olszyna et al. | 1998 | No | No | Yes | Yes | Yes |
| 25 | Panaphut et al. | 2002 | Yes | Yes | Yes | Yes | Yes |
| 26 | Parmar et al. | 2016 | Yes | Yes | Yes | Yes | Unclear |
| 27 | Perić et al. | 2005 | Yes | Yes | Yes | Yes | Unclear |
| 28 | Pertuiset et al. | 1988 | No | Yes | Yes | Yes | Yes |
| 29 | Phannajit et al. | 2023 | Yes | Yes | Yes | Yes | Yes |
| 30 | Philip et al. | 2021 | No | Yes | Yes | Yes | Yes |
| 31 | Raoult et al. | 1983 | Yes | Yes | Yes | Yes | Unclear |
| 32 | Rista et al. | 2022 | Yes | Yes | Yes | Yes | Yes |
| 33 | Sethi et al. | 2010 | Unclear | Unclear | Yes | Unclear | Unclear |
| 34 | Smith et al. | 2019 | Yes | Yes | Yes | Yes | Yes |
| 35 | Srisawat et al. | 2015 | Yes | Yes | Yes | Yes | Yes |
| 36 | Sukmark et al. | 2018 | Yes | Yes | Yes | Yes | Yes |
| 37 | Teles et al. | 2016 | Yes | Yes | Yes | Yes | Yes |
| 38 | Vidigal et al. | 2014 | Yes | Yes | Yes | Yes | Unclear |
| 39 | Wang et al. | 2018 | Yes | Yes | Yes | Yes | Yes |
| 40 | Yersin et al. | 1998 | Unclear | Yes | Yes | Yes | Yes |
| 41 | Fatema et al. | 2023 | Yes | Yes | Yes | Yes | Yes |
| 42 | Phannajit et al. | 2023 | Yes | Yes | Yes | Yes | Yes |
| 43 | Yang et al. | 2015 | Yes | Yes | Yes | Yes | Yes |
| 44 | Chang et al. | 2022 | Yes | Yes | Yes | Yes | Yes |

**Supplementary Figures**

**Supplementary Figure 1: Pooled frequency of Acute Kidney Injury in studies from South Asia**


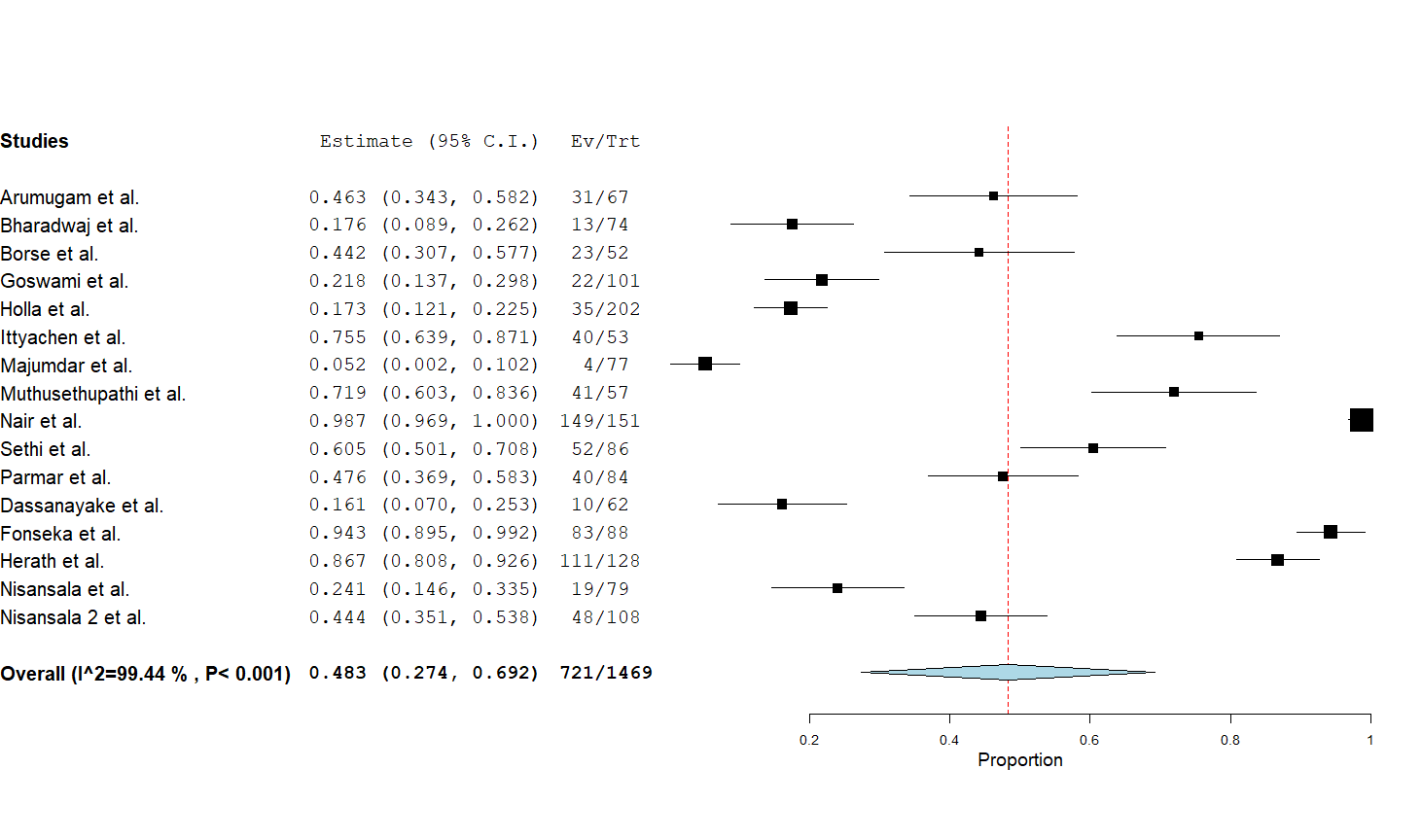


**Supplementary Figure 2: Pooled frequency of Acute Kidney Injury in studies from South-East Asia**


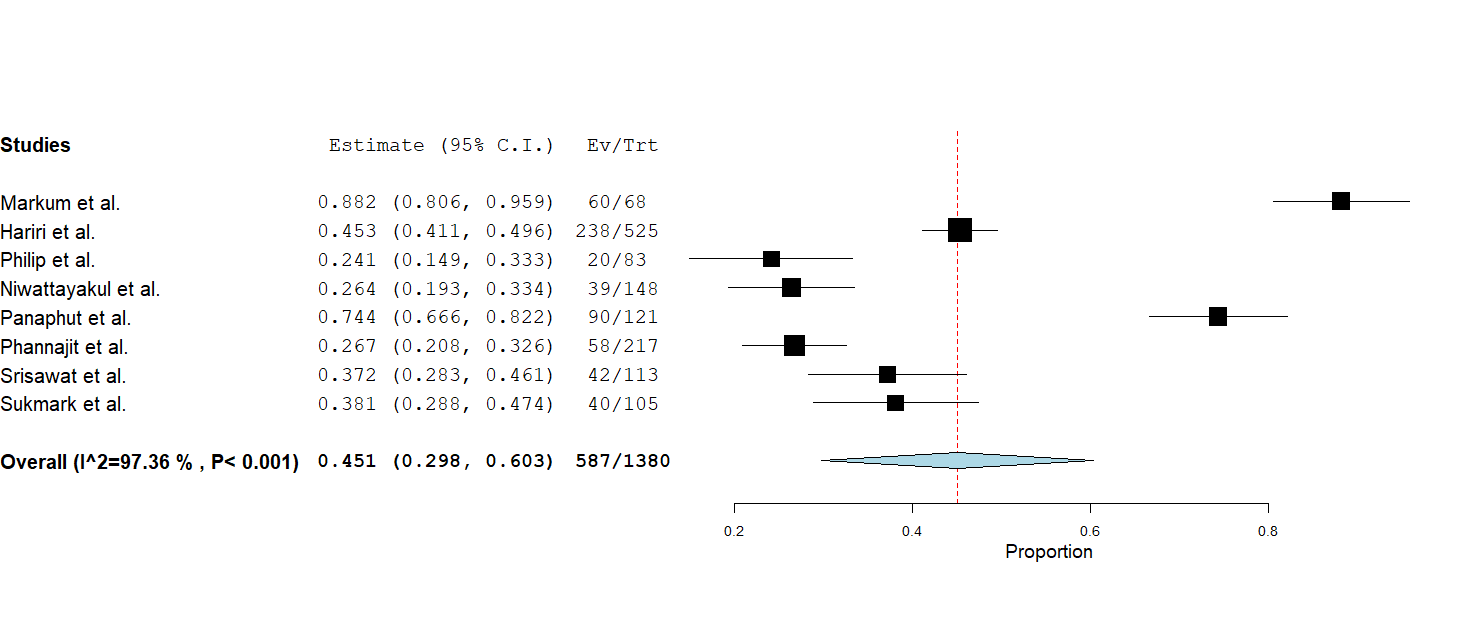


**Supplementary Figure 3: Pooled frequency of Acute Kidney Injury in studies from South America**


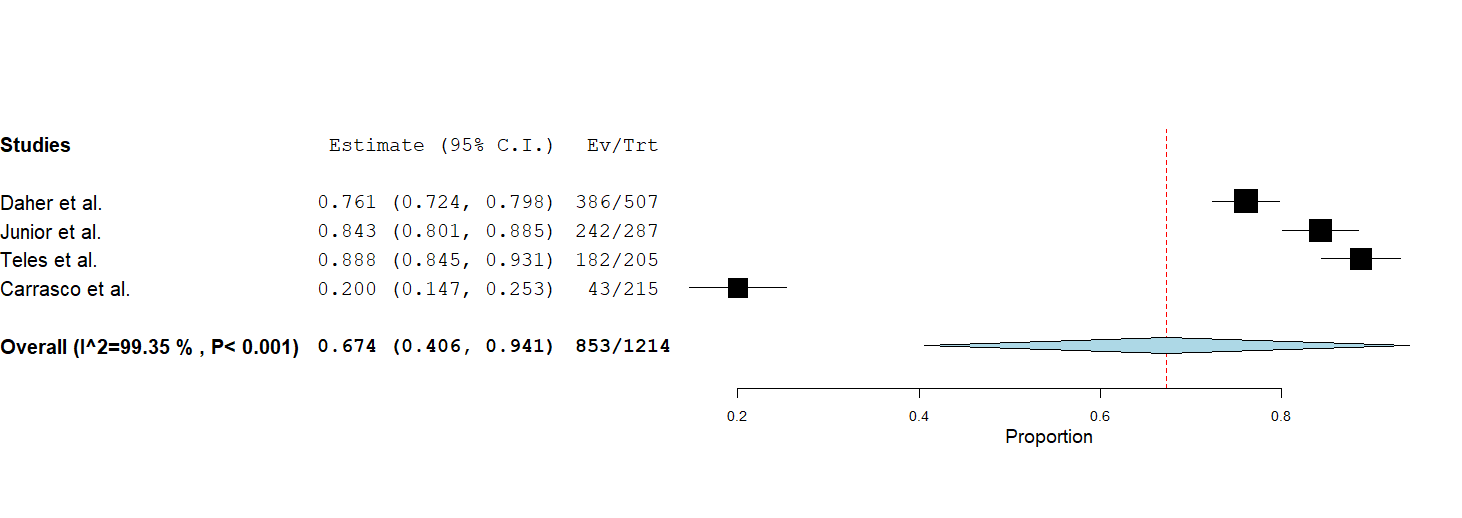


**Supplementary Figure 4: Pooled frequency of Acute Kidney Injury in studies from Europe**


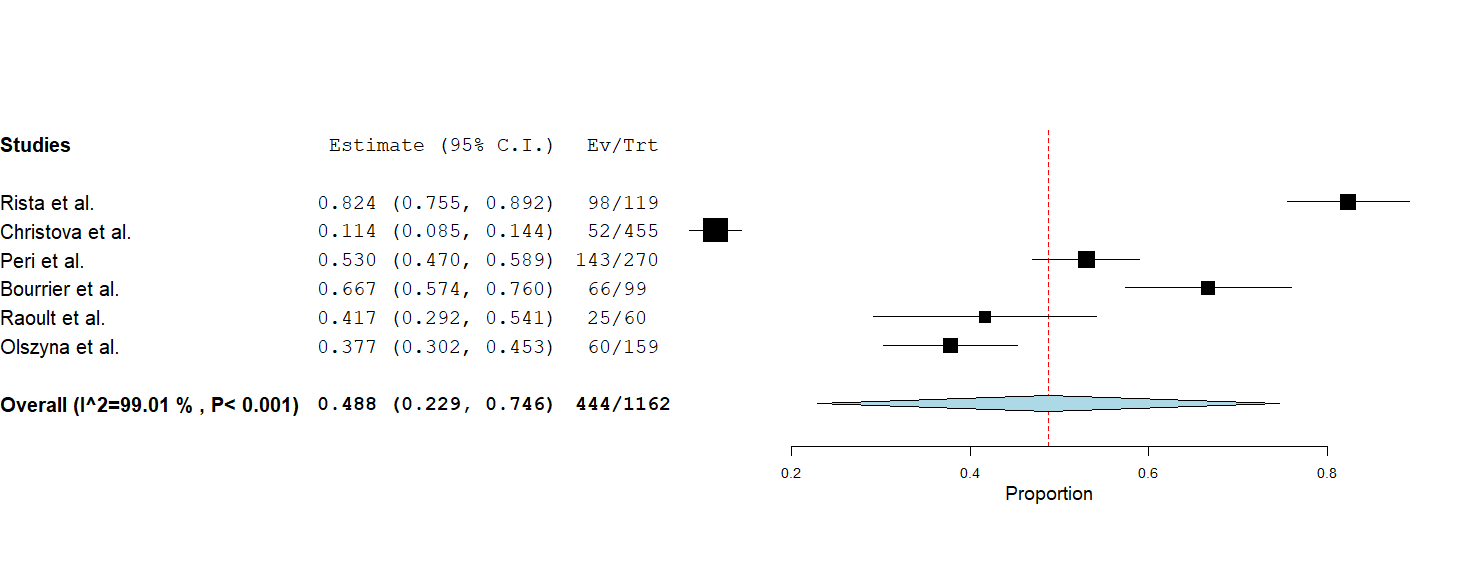


**Supplementary Figure 5: Pooled frequency of Acute Kidney Injury in studies that included patients of all severity**
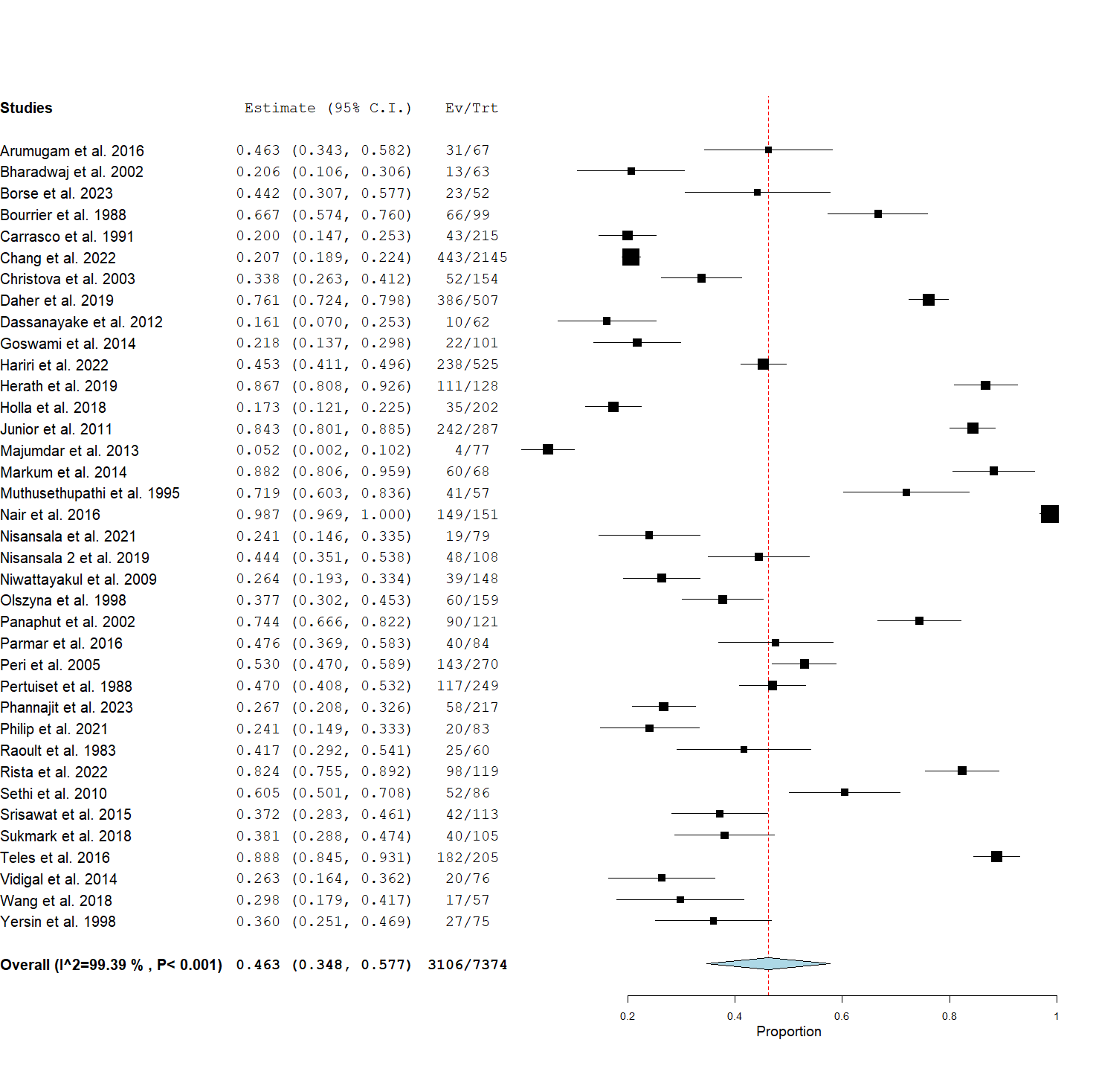


**Supplementary Figure 6: Pooled frequency of Acute Kidney Injury in studies that included only admitted patients** with leptospirosis
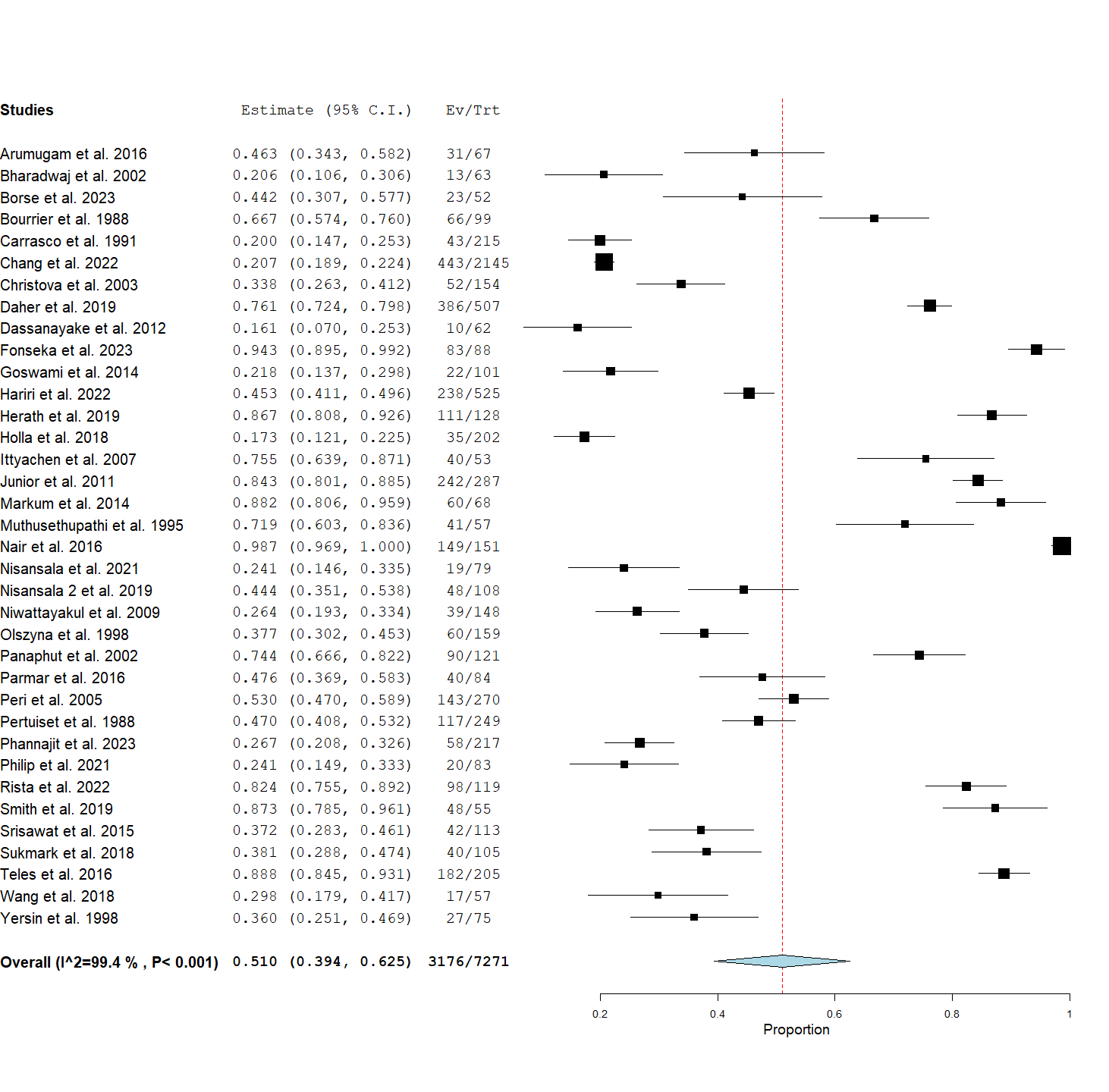


**Supplementary Figure 7: Pooled frequency of Acute Kidney Injury after subgroup analysis was done to include only those patients where a standardised definition (AKIN, RIFLE, KDIGO) was reported**


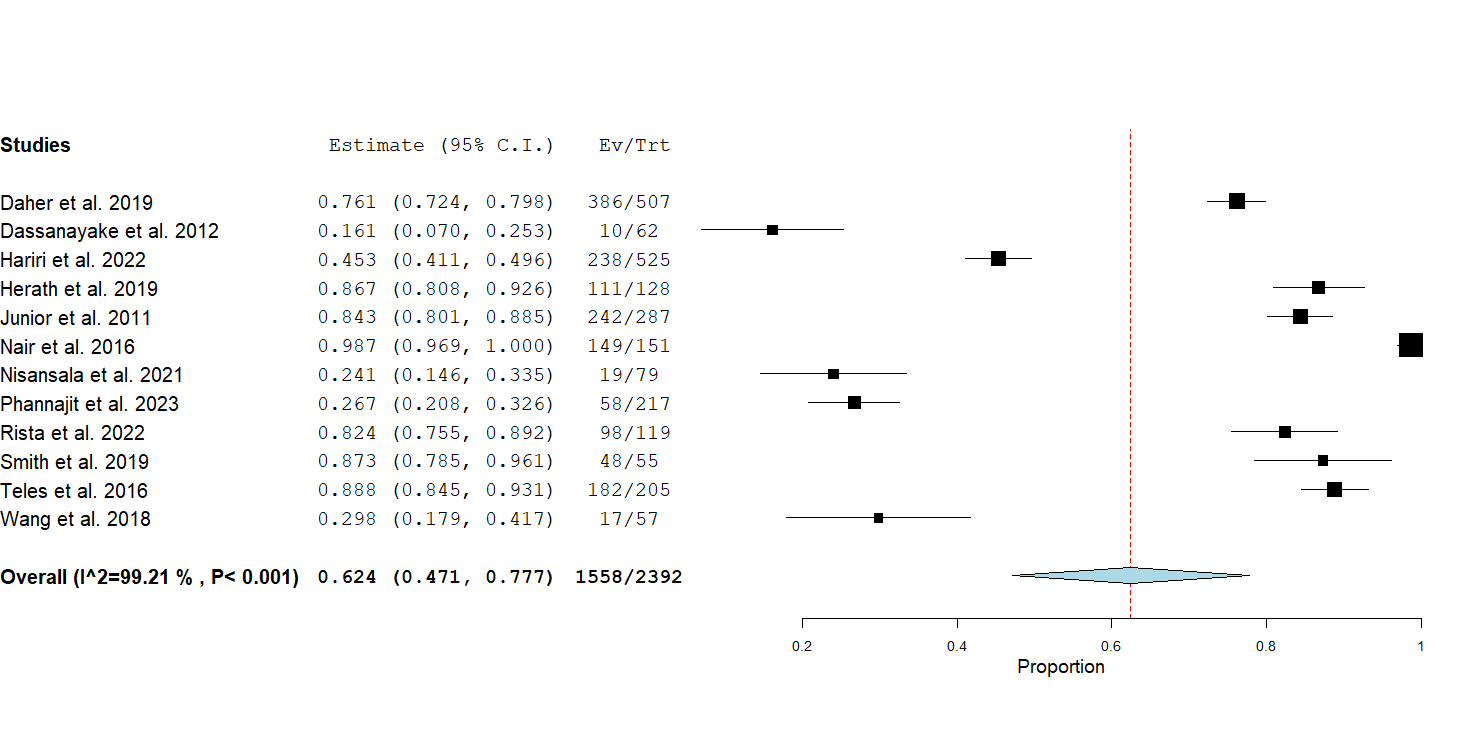


**Supplementary Figure 8: Pooled frequency of decreased urine output in patients with leptospirosis**


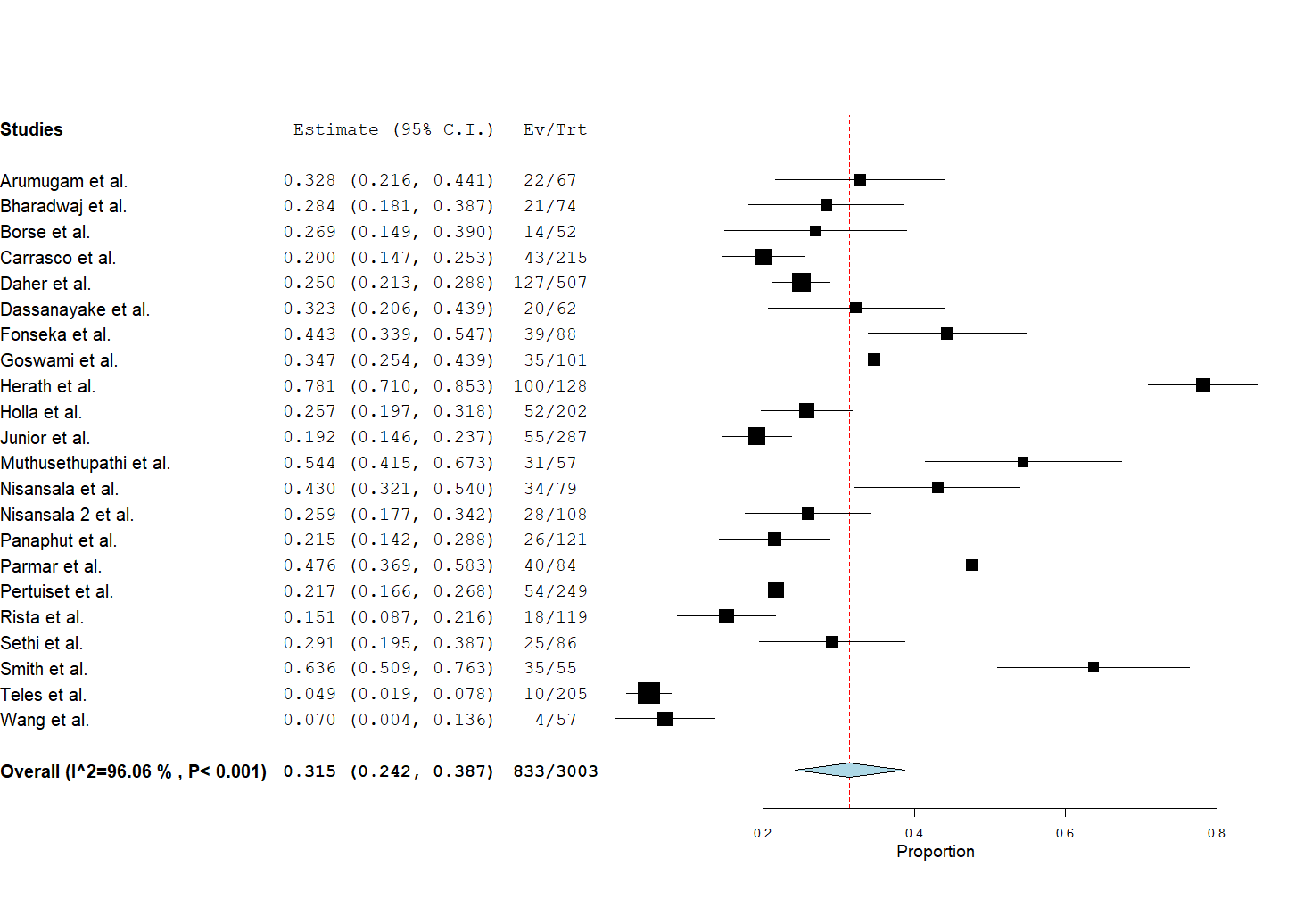


**Supplementary Figure 9: Pooled frequency of dialysis requirement in patients with leptospirosis**


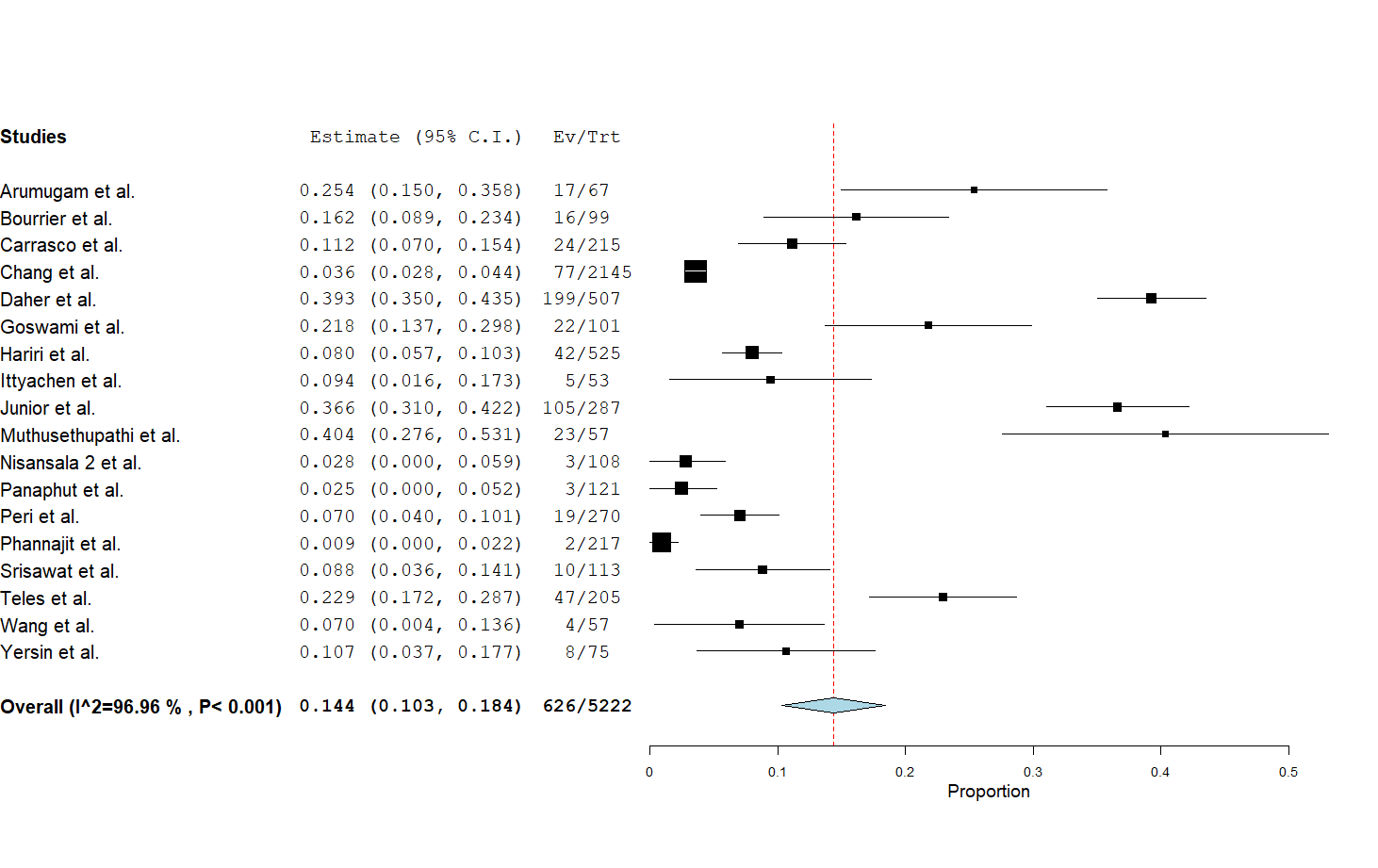


**Supplementary Figure 10: Pooled frequency of mortality in patients with leptospirosis**


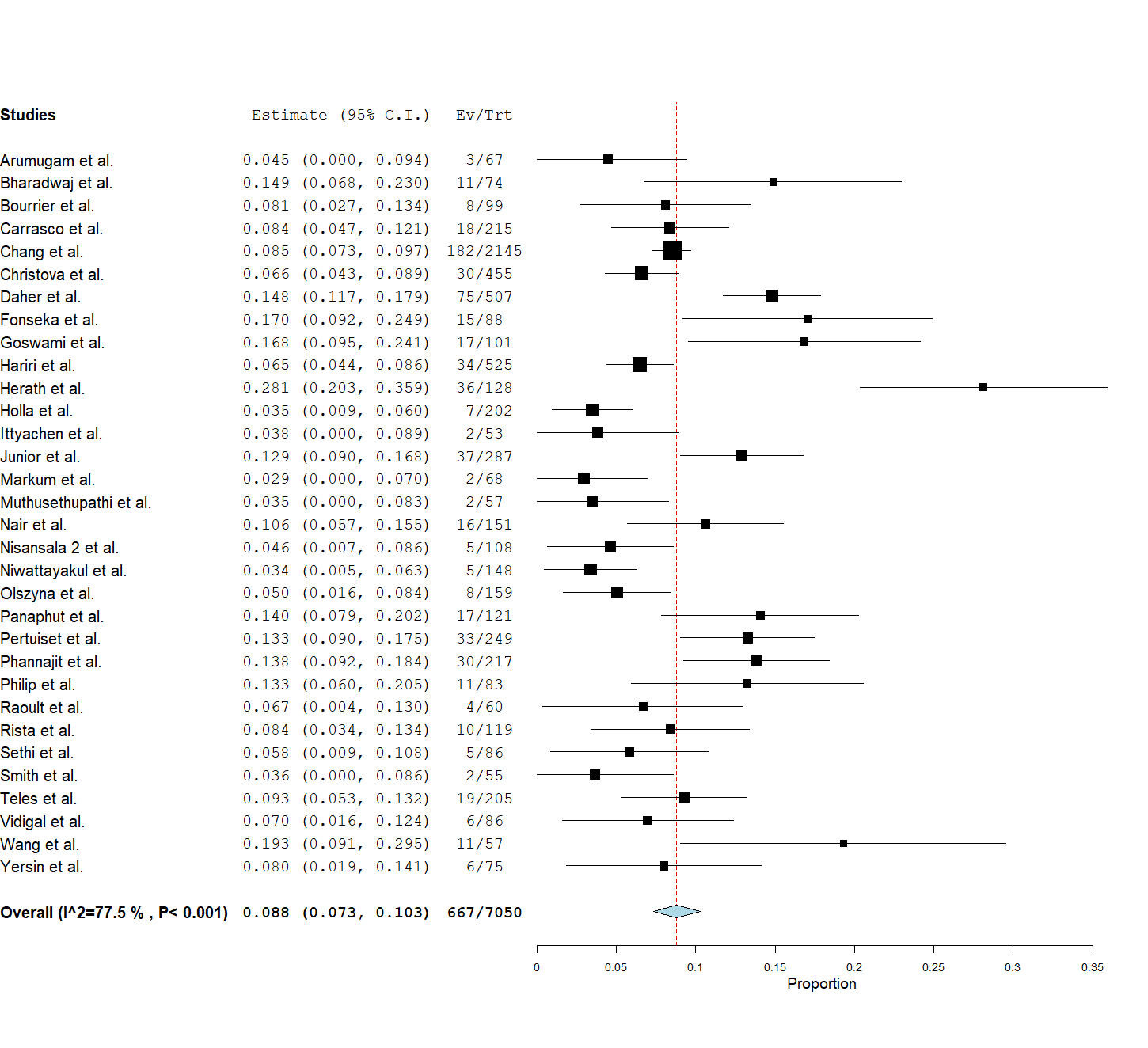
 **Supplementary Figure 11: Pooled frequency of mortality in patients with leptospirosis and Acute Kidney Injury**


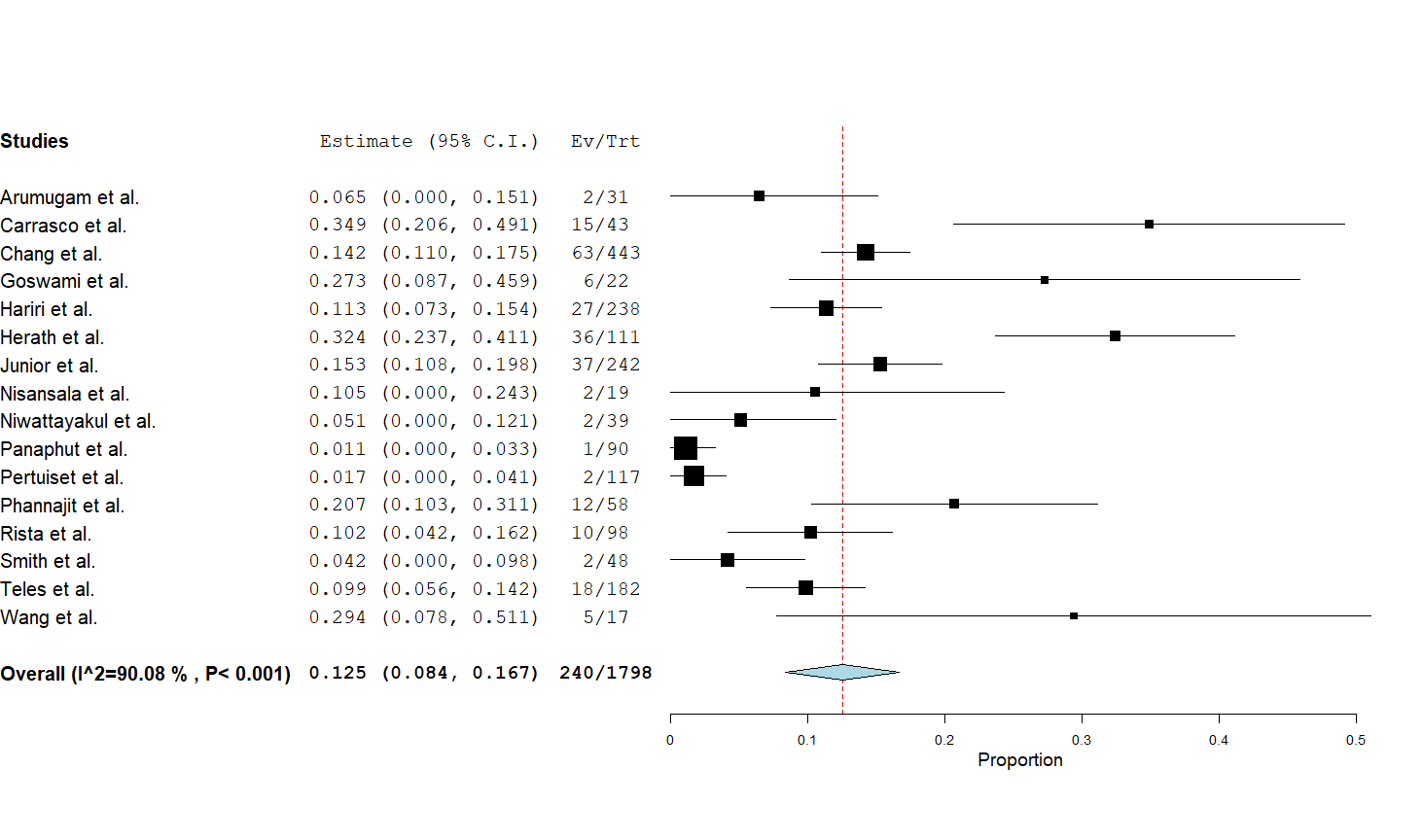


**Supplementary Figure 12: Pooled values of mean serum creatinine levels in patients with leptospirosis**


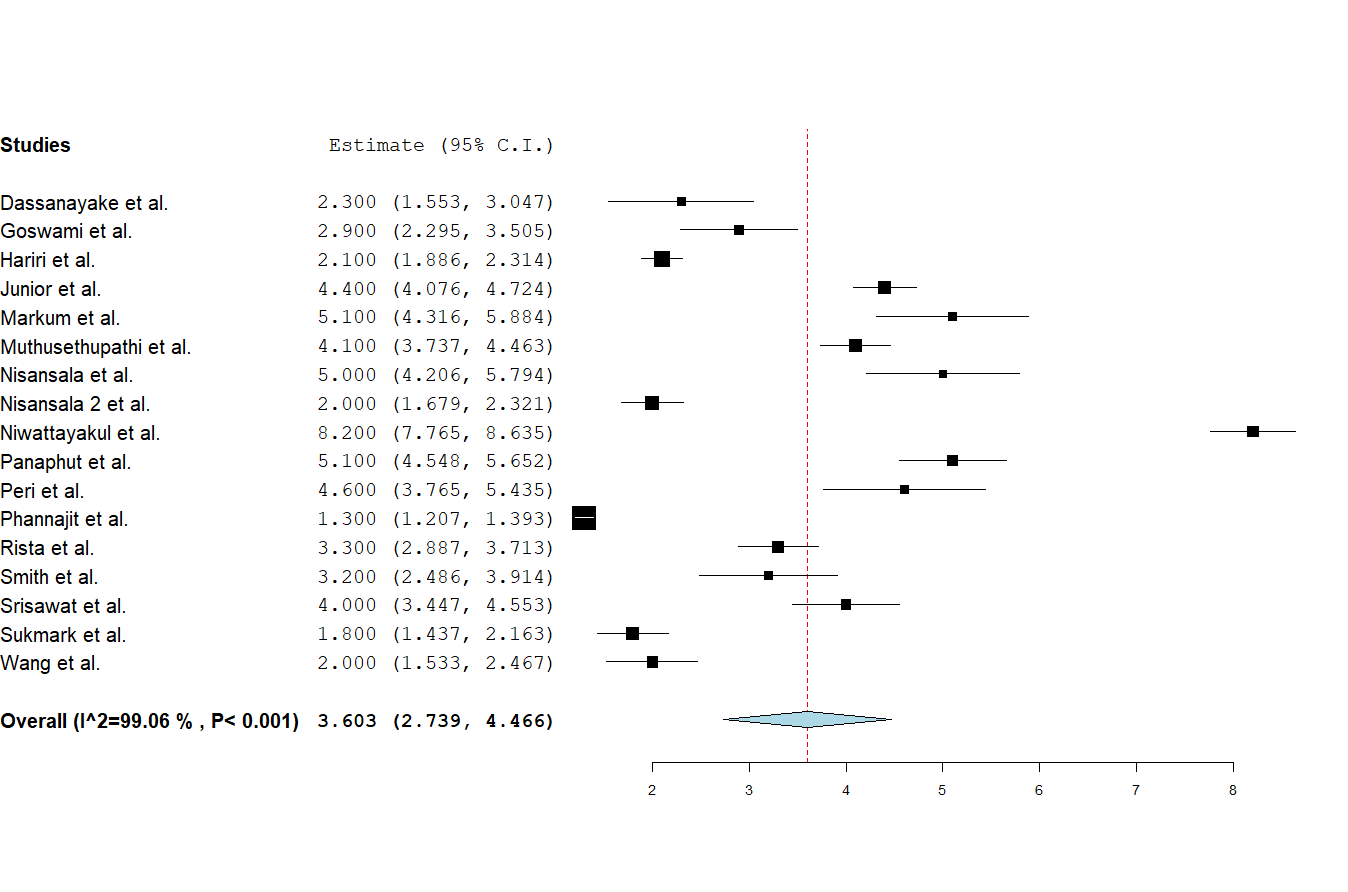


**Supplementary Figure 13: Pooled values of mean serum ureal levels in patients with leptospirosis**


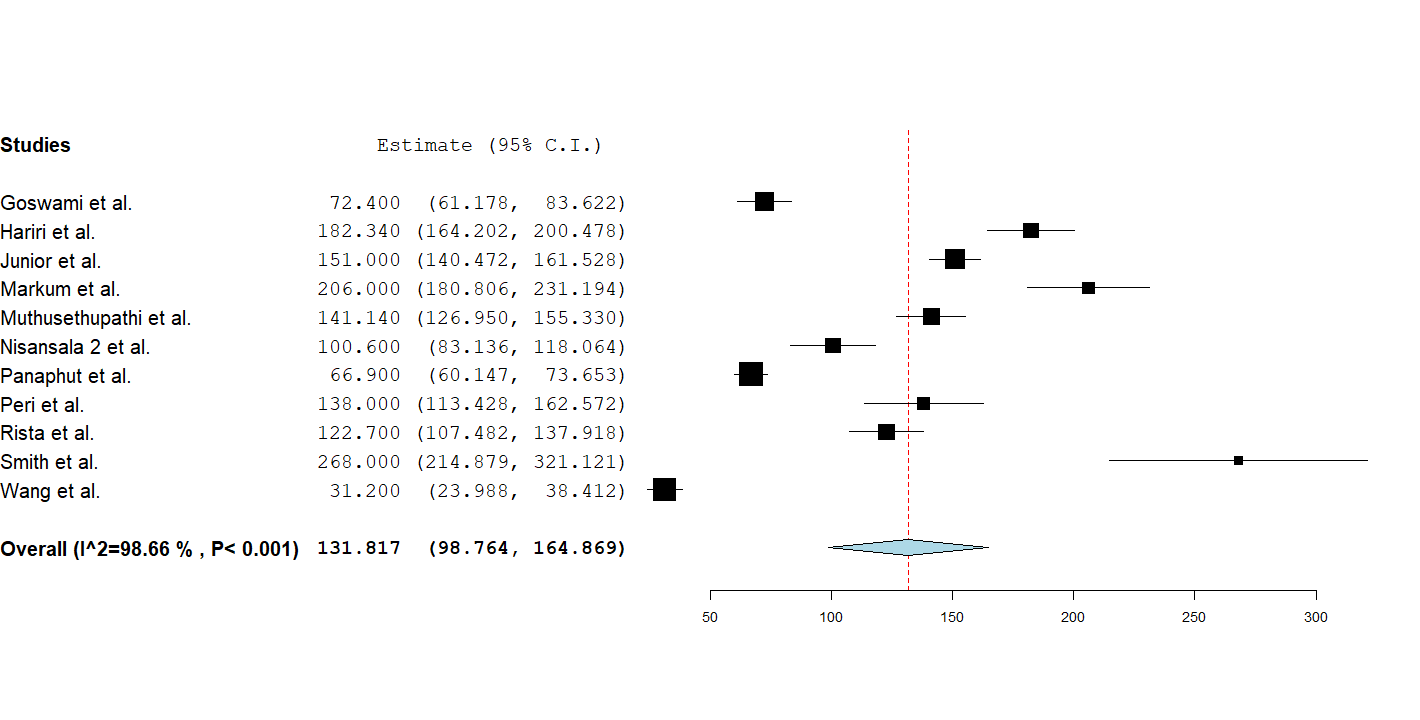

Supplement: Supplementary file 1 — Supplementary Material 1 [file 15010_2025_2492_MOESM1_ESM.docx]
